# The changes in chemical composition of *Holothuria tubulosa* (Gmelin, 1788) with ambient‐drying and oven‐drying methods

**DOI:** 10.1002/fsn3.703

**Published:** 2018-06-14

**Authors:** Şengül Bilgin, Hatice Öztürk Tanrikulu

**Affiliations:** ^1^ Egirdir Fisheries Faculty Suleyman Demirel University Isparta Turkey; ^2^ Graduate School of Applied and Natural Sciences Department of Fish Processing Technology Suleyman Demirel University Isparta Turkey

**Keywords:** drying technology, fatty acid, food composition, *Holothuria tubulosa*, sea cucumber

## Abstract

In this study, the nutritional properties of fresh (F), boiled (B), ambient‐dried (*Holothuria tubulosa*) (DA, 23 ± 2°C) and oven‐dried sea cucumber (DO, 45 ± 1°C) were compared in terms of proximate composition and fatty acid profiles. The results of the proximate analyses showed that the highest moisture content (86.76%) was determined in fresh samples, whereas the lowest moisture content (9.35%) was obtained in the oven‐dried group (DO). The crude fat and protein contents were in the range of 0.19% (B) to 0.87% (DA) and 12.30% (F) to 62.13% (DA), respectively. The highest ash content (30.30%) was obtained in group DO, while the lowest ash content (0.61%) was observed in the boiled samples (B). According to fatty acid analyses, there were no significant differences (*p *>* *0.05) between the two drying methods. The monounsaturated fatty acid (MUFA) (21.405%) and polyunsaturated fatty acid (PUFA) (36.018%) contents of *H. tubulosa* were high. *Holothuria tubulosa* can be used as protein and PUFA sources. Comparing the two methods, oven‐drying is better in terms of preservation, whereas drying at the ambient temperature is better in terms of nutrient value.

## 1 INTRODUCTION

Sea cucumbers, or their dried form, beche‐de‐mer, are a traditional delicacy prized by the Chinese and other Asian consumers for their dietary and curative properties (Robinson & Lovetelli, 2015). The use of Holothurians as a food began in China about 1,000 years ago (Purcell, Samyn & Conand, 2012). In China, more than 5,000 tons of dehydrated sea cucumbers are consumed every year (Bai, Qu, Luan, Li & Yang, 2013). The *S. herrmanni* species is widely utilized as a cure for asthma, hypertension, rheumatism, sinus, cuts, and burns in Malaysia (Fredalina et al., 1999).

Sea cucumber fisheries are currently employed in over 70 countries. Overexploitation is driving the most commercially valuable species to the risk of extinction. Overexploitation of sea cucumber resources therefore poses a threat to livelihoods, biodiversity, and the functioning of ecosystems (Robinson & Lovetelli, 2015). The latest estimates for the global annual catch of sea cucumbers are of the order of 100,000 metric tonnes (Purcell et al., 2012). The annual aquaculture production of sea cucumbers in China is 200,969 metric tonnes (Han, Keesing & Liu, 2016).

Sea cucumber aquaculture has shown a rapid increase in worldwide production. China remains the world leader in sea cucumber production. Juvenile cucumbers are intensively mass produced in recirculating aquaculture systems under controlled conditions (Robinson & Lovetelli, 2015). Also, sea cucumbers are excellent candidates as extractive organisms for co‐culture or integrated multi‐trophic aquaculture, as they feed principally on organically rich substrates, including waste from other species (Robinson & Lovetelli, 2015).

Many studies on holothuria have been carried out in other countries (Bechtel, Oliveira, Demir & Smiley, 2013; Chong, Pindi, Chye, Shaarani & Lee,^2015^; Duan, Zhang, Mujumdar & Wang, 2010; Omran, 2013; Pereira, Valentão, Teixeira & Andrade, 2013; Sicuro et al., 2012; Telahigue, Hajji, Imen, Sahbi & El Cafsi, 2014; Wen, Hu & Fan, 2010; Xu, Xu, Zhang, Peng & Yang, 2016), but there have been very few in Turkey (Aydın, 2016; Aydın, Sevgili, Tufan, Emre & Köse, 2011; Bilgin & İzci, 2016; Çaklı, Cadun, Kışla & Dinçer, 2004; Özer, Mol & Varlık, 2004).

Fresh sea cucumbers are not offered to market as much as the dried sea cucumbers are. Sun drying has been a traditional method for sea cucumbers since the ancient times as the energy required is free energy. However, this method is prone to pest invasions (Chong et al., 2015). Although *Holothuria tubulosa* is present in the Turkish Seas, it is not considered as a commercial species. This species has attracted attention because sea cucumbers are used in many countries as nutrients as well as in the health sector, and partly due to the fact that Turkey supplies this species. Therefore, the aim of this study was to determine the nutritional composition of fresh and dried *H. tubulosa,* and to compare the two drying methods used in drying the sea cucumber (oven‐drying and ambient‐drying).

## 2 MATERIALS AND METHODS

### 2.1 Materials

The *Holothuria tubulosa* (Gmelin, 1788) samples used in this study were harvested by divers from the İzmir Coast of the Aegean Sea, Turkey, in April. Scuba diving and snorkelling gear were used to harvest *H. tubulosa*. The internal organs of the sea cucumbers were removed by hand after their biometric measurements (weight, length, and diameter) had been taken. The cleaned sea cucumbers (100 individuals) were transported to the Food Processing Laboratory of the Egirdir Fisheries Faculty by cold chain in 4.5 hr. All 100 sea cucumbers were used, which had a mean length of 14.03 ± 4.63 cm, a mean weight of 68.97 ± 35.36 g and a mean diameter of 3.12 ± 0.68 cm.

### 2.2 Pretreatment

The sea cucumbers were boiled at 100°C for 20 min (Duan et al., 2010) and then placed on trays for cooling. The surface water was removed from the samples by filter paper. The biometric measurements of the boiled sea cucumbers were taken by dividing them into three groups at random with a similar weight in each group and labeled boiled (B); the other samples were similarly separated before drying in ambient temperature (DA) and by the oven (DO) drying process.

In the first of the two different drying processes used, *H. tubulosa* samples were placed on blotting paper without touching each other and then in a preheated oven (DO) at 45 ± 1°C for 76 hr. In the second method, the samples were again placed on blotting paper without touching each other and then put in an ambient temperature (DA). Ambient temperature was maintained at 23 ± 2°C. The samples were dried until they reached rock hardness (about 6 days). The sea cucumbers must be dried to rock hardness to prevent mold when they are stored, and to fetch a good price (Purcell, 2014). Once fully dried, the sea cucumbers were stored in large plastic sacks and kept in a dry place.

### 2.3 Proximate composition

Moisture content was determined with an automatic moisture analyzer (AND MX‐50). Crude protein content was determined according to the Kjeldahl method (N × 6.25) (Association of Official Analytical Chemists, 2000). Lipid content was determined by the Bligh and Dyer (1959) method, and the crude ash content was measured according to the Association of Official Analytical Chemists (2002).

### 2.4 Fatty acid analysis

Fatty acid methyl esters were extracted by transmethylation using a small amount of n‐heptane added to the lipids employing the method described by Ichihara, Shibahara, Yamamoto and Nakayama (1996). A 4 ml amount of 2M KOH was added to 10 mg lipid samples extracted with 2 ml of heptane. Afterward, the mixture was stirred with a vortex at an ambient temperature of 24 ± 2°C for 2 min and centrifuged at 4000 rpm for 10 min before the heptane layer was removed for gas chromatography (GC) analysis. Fatty acid methyl esters were separated by gas chromatography (PerkinElmer Clarus 500, USA) equipped with a flame ionization detector and a fused silica capillary SGE column (30 m × 0.32 mm, ID.BP20 0.25 μm, USA). The oven temperature was 140°C, held for 5 min, and then raised to 200°C at a rate of 1°C/min, while the injector and detector temperature were set to 220°C and 280°C, respectively. The sample size was 2 μl. The carrier gas was maintained at 16 psi, and the split rate was 1:100. Fatty acids were identified by comparing the retention times of FAME with a standard 37 component FAME mixture (Supelco). GC analyses were replicated twice. The results were calculated in the GC domain as % mean values ± standard error.

### 2.5 Statistical analysis

For statistical analysis, the results were expressed as mean ± standard error. One‐way analysis of variance (ANOVA) was carried out to determine the treatment effect (F, B, DA, and DO groups). Duncan's multiple comparisons test was used for comparison of the differences between averages. All statistic analyses were performed using the SPSS 16.0 software program (Esteves, 2011) (*p *= 0.05).

## 3 RESULTS AND DISCUSSION

### 3.1 Proximate composition

The effects of different treatments on the proximate compositions of *H. tubulosa* are given in Table 1. Similar to this study, Wen et al. (2010) reported that the moisture content was 11.6% and 7.0% in dried *Holothuria fuscogilva* and *Holothuria fuscopunctata* species, respectively. Özer et al. (2004) indicated that the moisture content of fresh *Holothuria scabra* was determined to be 87.21%, 85.45%, 84.91%, 85.32%, and 84.54% from April to August, respectively. Similarly, Aydın et al. (2011) found the moisture content to be 84.30% in fresh *H. tubulosa*. Zhong, Khan and Shahidi (2007) evaluated proximate and fatty acid compositions of fresh and rehydrated sea cucumber (*Cucumaria frondosa*) samples with/without internal organs. The moisture content of fresh *C. frondosa* with/without internal organs was determined to be 90.1/87.4%. Çaklı et al. (2004) determined that the moisture content of *H. tubulosa* was 86.74% when raw, 66.17% when boiled and 20.47% in dried samples (8 days). However, the moisture content of boiled and dried *H. tubulosa* was different from this study. The reason for these differences may be the boiling and drying conditions. Bechtel et al. (2013) reported a moisture content of 4% in freeze‐dried samples of *Parastichopus californicus*. Bilgin and İzci (2016) found that the moisture significantly decreased in dried samples (10.33%) compared to fresh (86.93%) and boiled *H. forskali* (80.82%). Similar findings were found in this study.

**Table 1:** Proximate compositions of muscle of *Holothuria tubulosa* (%means ± SE)

Proximate composition	F	B	DA	DO
Moisture	86.760 ± 0.071^a^	81.660 ± 0.928^b^	10.350 ± 0.005^c^	9.347 ± 0.014^c^
Crude protein	12.300 ± 0.212^c^	17.843 ± 167^b^	62.127 ± 1.561^a^	60.956 ± 0.303^a^
Crude fat	0.221 ± 0.014^b^	0.191 ± 0.001^b^	0.873 ± 0.091^a^	0.758 ± 0.053^a^
Crude ash	0.723 ± 0.003^c^	0.610 ± 0.034^c^	26.650 ± 0.025^b^	30.300 ± 0.413^a^

*Notes*

DA: dried at ambient temperature; DO: dried in the oven; F: Fresh; B: Boiled.

In same line, means with different letters are significantly different (*p *<* *0.05).

In this study, the highest crude protein rate (62.127%) was found in the DA group, while the lowest protein rate (12.30%) was seen in the F group (*p *< 0.05). In addition, differences among the F, B, and dried groups were found to be statistically significant (*p *< 0.05). Similarly, Çaklı et al. (2004) reported that the crude protein rate was 8.18% in fresh, 15% in boiled, and 66.45% in completely dried sea cucumbers. Aydın et al. (2011) detected an 8% protein content for fresh *H. tubulosa*. Bilgin and İzci (2016) found that the protein content significantly increased in dried samples (60.92%) by comparison with fresh (11.99%) and boiled *H. forskali* (17.25%) In that study, the sea cucumbers were processed using two different methods. The first method was evisceration by cutting the anus, followed by the removal of the internal organs by firmly squeezing the body; the second method was through evisceration by cutting along the length of the body, followed by the removal of the internal organs (Özer et al., 2004). They indicated that crude protein rates were determined to be 59.57% in method 1 in April and 60.18% in method 2 in April for *H. scabra*. Chang‐Lee, Price and Lampila (1989) reported the protein content to be 61.70% in dried sea cucumbers. Telahigue et al. (2014) indicated improvements in the total protein level in the body wall of *H. forskali* dried at different temperatures and humidity levels. Compared to each other, an increase in the protein contents was observed to be dependent on a decrease in the moisture content in these studies. Similar results were found in this study. Bechtel et al. (2013) reported 68% protein in the muscle bands, and 47% in the body wall of freeze‐dried *P. californicus*.

In this study, the crude fat contents showed significant changes among groups (*p *< 0.05). However, there was no significant difference in the crude fat rates between F and B (0.221%–0.191%) and between DA and DO (0.873%–0.758%) groups (*p *>* *0.05). Similar to this study, Özer et al. (2004) indicated that crude fat rates were determined to be 0.37, 0.22, 0.19, 0.17, and 0.23% in fresh *H. scabra* species in April, May, June, July, and August, respectively. Contrary to this study, Çaklı et al. (2004) reported that in *H. tubulosa* species, crude fat content was 0.16% in fresh, 0.41% in boiled, and 0.60% in completely dried samples. Zhong et al. (2007) indicated that the crude fat contents found in fresh (*Cucumaria frondosa*) samples with/without internal organs were 0.50%–0.70% and 1.16%–1.27% in processed products, respectively. Wen et al. (2010) determined that crude fat was 0.3% in both dried *H. fuscogilva* and *H. fuscopunctata*. Chang‐Lee et al. (1989) indicated an 0.3% lipid content in fresh samples and 2%–3% in dried samples. Bechtel et al. (2013) reported 5% lipids in muscle bands, 26% ash, and 8% lipids in the body wall of freeze‐dried *P. californicus*. Aydın et al. (2011) detected 0.18% lipid content in fresh *H. tubulosa*. Bilgin and İzci (2016) found that the lipids significantly increased in dried *H. forskali* (from 0.256% to 0.866%*)*. Differences in crude fat contents may be caused by processing conditions, hunting time, and species variety.

In this study, the crude ash contents were significant among groups (*p *< 0.05). However, there was no significant difference in the crude ash rates between F and B (0.72% and 0.61%) groups (*p *>* *0.05). The ash contents in dried samples were determined to be 26.65% for DA, and 30.30% for DO. Similarly, Wen et al. (2010) determined that ash contents were 26.4% and 39.6% in dried *H. fuscogilva* and *H. fuscopunctata* species, respectively. By comparison, the amount of ash increased due to the drying procedure in both studies. On the contrary, Zhong et al. (2007) indicated that the crude ash contents were found to be 2.97% and 3.03% in fresh *Cucumaria frondosa* samples with/without internal organs. Özer et al. (2004) indicated that crude ash rates were determined to be 8.10, 11.06, 7.57, 4.68, and 3.59% in fresh *H. scabra* species in April, May, June, July, and August, respectively. Aydın et al. (2011) detected a crude ash content of 5.13% in fresh *H*. *tubulosa*. Bechtel et al. (2013) reported 2% ash in the muscle bands, and 26% in the body wall of freeze‐dried *P. californicus*. Bilgin and İzci (2016) found that the ash value significantly increased in dried *H. forskali* (from 0.736% to 27.34%*)*. Those results agreed with the results of this study (Table 1).

### 3.2 Fatty acid profile

The fatty acid profiles of all *H. tubulosa* samples (all groups) are given in Table 2. In this study, PUFA (36.018%) was found to be higher than the SFA (19.730%) and MUFA (21.405%) contents of fresh *H. tubulosa*. The total MUFA contents of boiled (17.993%) and DA samples (16.320%) decreased compared to the fresh samples (21.405%). The total PUFA content increased in the DA samples (41.773%), whereas it decreased in the DO samples (35.515%). The total SFA contents (19.730% in F group) decreased in the DA samples (16.443%). Bechtel et al. (2013) reported PUFA contents as 28.88% and 43.64% in freeze‐dried samples of the body wall and muscle bands of *P. californicus*. Bechtel et al. (2013) reported MUFA contents to be 39.80% and 32.05% in freeze‐dried samples of the body wall and muscle bands of *P. californicus*. Bilgin and İzci (2016) indicated that EPA, ARA, and PUFA contents significantly decreased in boiled and dried samples of *H. forskali*, while the SFA content decreased after the boiling process compared to the fresh samples. Telahigue et al. (2014) indicated improvements in the PUFA level in the body wall of *H. forskali* dried at different temperatures and humidity levels. Bechtel et al. (2013) reported SFA contents to be 26.94% and 18.13% in freeze‐dried samples of the body wall and muscle bands of *P. californicus*.

**Table 2:** Fatty acid profiles in meat of *Holothuria tubulosa* (% means ± SE)

FA types	F	B	DA	DO
C12:0	0.152 ± 0.004^b^	0.185 ± 0.009^a^	0.030 ± 0.017^c^	0.305 ± 0.002^a^
C14:0	1.652 ± 0.053^a^	1.092 ± 0.042^b^	0.220 ± 0.004^e^	1.490 ± 0.063^ab^
C16:0	6.460 ± 0.574^a^	3.617 ± 0.201^cd^	1.330 ± 0.060^e^	6.475 ± 0.060^a^
C17:0	0.320 ± 0.010^c^	0.450 ± 0.030^b^	0.207 ± 0.073^d^	0.550 ± 0.005^a^
C18:0	7.205 ± 1.017^bc^	11.285 ± 0.143^a^	11.682 ± 0.577^a^	10.915 ± 0.083^ab^
C20:0	1.420 ± 0.100^def^	1.527 ± 0.133^cdef^	1.420 ± 0.012^def^	0.575 ± 0.002^d^
C23:0	1.085 ± 0.148^ab^	0.580 ± 0.060 ^cd^	1.227 ± 0.101^a^	1.055 ± 0.014^a^
C24:0	1.435 ± 0.137^b^	0.915 ± 0.023 ^cd^	0.325 ± 0.009^e^	0.745 ± 0.002^bcd^
∑SFA	19.730 ± 1.470^ab^	19.652 ± 0.262^ab^	16.443 ± 0.623^bc^	22.110 ± 0.225^a^
C14:1	0.800 ± 0.033^ab^	0.647 ± 0.033^bc^	0.145 ± 0.014^e^	1.055 ± 0.002^a^
C16:1	4.800 ± 0.197^a^	2.730 ± 0.150^c^	0.865 ± 0.009^f^	4.550 ± 0.040^b^
C17:1cis‐10	0.160 ± 0.035 ^cd^	0.240 ± 0.072 ^cd^	0.340 ± 0.135^bc^	0.435 ± 0.002^c^
C18:1 ω‐9	4.062 ± 0.334^bc^	3.355 ± 0.046^cde^	2.200 ± 0.087^efg^	5.885 ± 0.054^a^
C18:1 n7	2.517 ± 0.074 ^cd^	1.160 ± 0.081^f^	1.042 ± 0.011^f^	1.495 ± 0.014^ef^
C20:1	6.900 ± 0.240^bc^	8.445 ± 0.056^b^	11.232 ± 0.142^a^	7.535 ± 0.054^bcd^
C22:1 ω‐9	1.487 ± 0.091^b^	1.107 ± 0.464^bc^	0.297 ± 0.086^de^	0.770 ± 0.017^e^
C24:1	0.645 ± 0.048^a^	0.307 ± 0.083^bc^	0.197 ± 0.067^c^	‐
∑MUFA	21.405 ± 0.372^a^	17.993 ± 0.962^bcd^	16.320 ± 0.453^e^	21.725 ± 0.153^abc^
C18:2 ω‐6	4.020 ± 0.110^a^	3.665 ± 0.050^ab^	3.232 ± 0.172^ab^	2.945 ± 0.031^abc^
C18:3 ω‐3	1.012 ± 0.106^bc^	0.832 ± 0.018 ^cd^	0.850 ± 0.005 ^cd^	0.645 ± 0.014^e^
C20:2 cis	0.625 ± 0.133^bcde^	0.912 ± 0.027^b^	1.565 ± 0.181^a^	1.365 ± 0.020^a^
C20:4 ω‐6	17.167 ± 0.594^bc^	20.467 ± 0.165^b^	25.352 ± 0.296^a^	19.020 ± 0.150 ^cd^
C20:5 ω‐3	10.027 ± 0.385 ^cd^	9.597 ± 0.065^d^	6.767 ± 0.234^e^	9.135 ± 0.106^c^
C22:6 ω‐3	3.165 ± 0.139^cde^	2.845 ± 0.079^de^	4.005 ± 0.124^abc^	2.405 ± 0.014 ^g^
∑PUFA	36.018 ± 0.765 ^cd^	38.320 ± 0.247^bc^	41.773 ± 0.634^a^	35.515 ± 0.338^bcd^

*Notes*

DA: dried at ambient temperature; DO: dried in the oven; F: Fresh; B: Boiled.

In same line, means with different letters are significantly different (*p *<* *0.05).

EPA and DHA contents showed a decrease after the boiling and drying process (Table 2). Wen et al. (2010) indicated that DHA was not found in some dried samples, and the reason may be the repetitive boiling process. It has also been reported that long‐chain fatty acids such as EPA and DHA may be highly susceptible to heating and other culinary processes (Wen et al., 2010). These results were similar to our findings. Fredalina et al. (1999) reported that the DHA ratio was found to be higher than EPA in extractions with water of *Stichopus chloronotus* using different methods. In *H. tubulosa,* the opposite situation was found. Zhong et al. (2007) determined high EPA and DHA ratios in fresh samples, fresh samples with internal organs, rehydrated samples with internal organs, and rehydrated samples without internal organ in *C. frondosa*. On the contrary, ARA forms of PUFA were found to be high in this study. A similar result was reported by Svetashev et al. (1991) in 12 Holothurian species. Aydın et al. (2011) detected a decrease in DHA and PUFA, and an increase in ARA and EPA after drying *H. tubulosa*. Bechtel et al. (2013) reported 6.19% and 8.93% DHA, and 12.34% and 22.63% EPA contents in the body wall and muscle bands of freeze‐dried *P. californicus*, respectively. The same researchers reported ARA contents to be 7.05% and 9.90% in freeze‐dried samples of the body wall and muscle bands of *P. californicus*. Pereira et al. (2013) reported that EPA–DHA rates were low, and the PUFA contents were higher than SFA in freeze‐dried samples of *H. forskali* caught from Portugal.

According to fatty acid analyses, lower MUFA and PUFA values were found in samples dried at room temperature. This situation may be due to the fact that the fatty acids were oxidized when the product was dried at room temperature. Palmitic acid, palmitoleic acid, and oleic acid decreased significantly after drying under (DA) conditions for *H. tubulosa*. It is known that fatty acid components may vary according to processing, storage, and body parts (Bilgin, 2003; Wen et al., 2010).

## 4 CONCLUSION

*Holothuria tubulosa* was found to have low fat contents (0.221%) and can be considered as a dietetic food. MUFA (21.405%) and PUFA (36.018%) contents of *H. tubulosa* were higher than SFA (19.730%) in fresh samples. The *H. tubulosa* species was rich in terms of PUFA, especially ARA (17.167%) and EPA (10.027%), which are significant for nutrition. Drying temperatures (23 ± 2°C for about 6 days and 45 ± 1°C for 76 hr) can decrease the essential fatty acid (EPA and DHA) content of *H. tubulosa*. The total MUFA significantly decreased in the DA groups. The drying methods can change the nutritional contents of *H. tubulosa*. Oven‐drying at 45 ± 1°C, or drying at ambient temperature (23 ± 2°C), slightly affected the essential fatty acids. The drying process caused an increase in some fatty acids, such as ARA, myristoleic acid and stearic acid, and a decrease in some fatty acids such as linoleic acid, linolenic acid, EPA. This is a result of many factors, such as fat oxidation, nutrition, and individual differences. For this reason, such studies should be supported by more sampling.

Oven‐drying treatment yielded the highest SFA (22.110%) and MUFA (21.725%) contents when compared to those of the other samples. EPA, DHA, and total PUFA contents of *H. tubulosa* decreased after the oven‐drying process. EPA, DHA, and total PUFA are long chain fatty acids and may be oxidized easily. Weber, Bochi, Ribeiro, Victo and Emanuelli (2008) have stated that major changes during processing of heated food are associated with oxidation. PUFAs (i.e., EPA & DHA) are considered to be susceptible to oxidation during heating. This information supports our results. According to results of the fatty acid analyses, if the two drying methods are compared in terms of their fatty acid compositions; as an advantage for oven‐drying, the total PUFA, DHA, and ARA contents were higher in DA group than those in DO group. Furthermore, ambient conditions have uncontrolled atmosphere. This is a disadvantage for ambient‐drying as the hygienic conditions and ambient temperature, etc. will be harder to control. Dried sea cucumbers in ambient temperature are more prone to oxidation than they are under oven‐drying conditions due higher exposure to oxygen and light. These conditions affect the quality of end products. In terms of proximate composition, oven‐dried sea cucumbers have lower moisture and fat contents. Therefore, it is thought that oven‐drying provided a better protection. The ash content was higher in oven‐dried sea cucumbers. Also, protein ratio was lower than those dried at ambient temperature. In conclusion, drying *H. tubulosa* under controlled conditions, such as oven‐drying, cabinet drying is a better method for healthy consumption.

## CONFLICT OF INTEREST

The authors declare no conflicts of interest.

## DECLARATION

This study has nothing to do with human and animal testing.
